# Effect of Regularization on Efficient Modeling and Simulation of Bioinspired Composites Using Cohesive Zone Method

**DOI:** 10.3390/biomimetics11020139

**Published:** 2026-02-12

**Authors:** Md Jalal Uddin Rumi, Xiaowei Zeng

**Affiliations:** Department of Mechanical, Aerospace & Industrial Engineering, University of Texas at San Antonio, San Antonio, TX 78249, USA; mdjalaluddin.rumi@utsa.edu

**Keywords:** bioinspired composites, cohesive zone modeling, polyhedral mesh regularization, finite element fracture simulation, Voronoi tessellation

## Abstract

Tessellation-based polyhedral microstructures derived from Voronoi and Laguerre constructions provide a realistic geometric foundation for modeling bioinspired organic–inorganic composites with interfacial fracture. However, even after extensive centroidal relaxation, such tessellations retain numerous lower-dimensional geometric degeneracies—very short edges and small or sliver-like faces—that severely hinder volumetric meshing and render large-scale cohesive-zone simulations computationally impractical. In this work, we employ a geometric regularization step that enforces a minimum admissible feature length prior to meshing and systematically quantify its impact on downstream performance in finite element discretization and cohesive fracture simulation. By eliminating geometric features below the prescribed length scale while preserving grain topology and morphology, the regularized tessellations exhibit sharply improved edge-length and face-diameter distributions and become readily meshable at practical resolutions. When applied to a 3D bioinspired organic–inorganic composite with cohesive interfaces, the regularized geometry reduces volumetric and cohesive element counts nearly fivefold and increases the explicit stable time increment by approximately four orders of magnitude, transforming an otherwise diverging analysis into a robust simulation that converges to the prescribed deformation. These results demonstrate that the prescribed geometric regularization step is not merely a preprocessing refinement but a critical enabling step for efficient and large-scale cohesive fracture simulations of tessellation-based bioinspired composites.

## 1. Introduction

The mechanical response of heterogeneous materials is governed by their microstructure, including the morphology and spatial arrangement of constituent phases and the nature of the interfaces between them [1,2,3,4]. Advances in 3D characterization have enabled detailed descriptions of real polycrystalline morphologies, including grain size distributions, neighborhood statistics, and boundary curvatures [5,6,7]. In parallel, the finite element method (FEM) has been widely used to simulate the behavior of virtual polycrystals [8,9,10,11]. Many early models employed idealized grain shapes (e.g., cubes, dodecahedra, truncated octahedra) that are straightforward to mesh but only coarsely represent realistic microstructures [12,13,14,15]. Voronoi and Laguerre tessellations offer a more faithful alternative by generating analytically defined polyhedral grains with planar boundaries and straight triple lines [16]. However, their geometric irregularity makes high-quality mesh generation challenging; thus, free meshing of tessellation-based microstructures has often been restricted to relatively small systems (fewer than ∼10^3^ grains) and modest deformation levels [17,18,19,20,21], where extreme element distortion is less likely to arise.

Bioinspired composites such as nacre, bone, and dental enamel achieve exceptional combinations of stiffness, strength, and toughness by combining stiff mineral constituents (e.g., aragonite or hydroxyapatite) with a compliant organic matrix in hierarchical architectures [22,23,24,25,26,27,28,29]. At the mesoscale, these materials can be effectively represented as polyhedral grains bonded by soft interfaces, enabling explicit capture of mechanisms such as interface sliding, delamination, crack deflection, and distributed interfacial damage. Tessellation-based constructions, particularly Voronoi tessellations and centroidal Voronoi tessellations (CVTs), provide a natural geometric basis for generating such grain structures [30,31,32]. Coupled with cohesive zone modeling (CZM) at grain boundaries, these representations have been used to investigate fracture and toughening mechanisms in bioinspired composites [27,32,33,34,35]. Nonetheless, as model size and deformation increase, the tessellation geometry itself often becomes the primary bottleneck for robust and efficient simulation.

A key difficulty is that tessellation-based polyhedral models commonly contain undesirable small features such as very short edges, small or sliver-like faces, and thin polyhedral entities, which severely degrade downstream finite element (FE) meshing and analysis. These features lead to highly distorted tetrahedra, restrict stable time steps in explicit dynamics, and can prevent reliable insertion of cohesive interfaces along grain boundaries. Many existing studies sidestep these issues by limiting the number of grains or the applied deformation, or by relying on ad hoc mesh refinement and smoothing [36,37,38]. However, such approaches operate on an already-generated volumetric mesh, and as such cannot eliminate geometric degeneracies that originate at the tessellation level, often causing volumetric meshing to fail at the desired resolution. More recently, the virtual element method (VEM) has enabled formulations on general polyhedral meshes [39,40]. While VEM alleviates some meshing constraints, cohesive-zone modeling still requires explicit and well-conditioned interface discretization, for which uncontrolled lower-dimensional features remain detrimental. In this context, geometry-level regularization via controlled edge collapse operations is particularly effective, as it removes problematic features prior to meshing and directly targets the source of numerical instability in large-scale CZM simulations.

Motivated by these limitations, this work employs a geometric regularization step for tessellation-based microstructures and investigates how enforcing a controllable minimum feature length improves practical modeling and simulation of bioinspired organic–inorganic composites with CZM. The prescribed regularization eliminates geometric features below a user-defined threshold through local topology-preserving collapse operations while maintaining the conforming grain connectivity and overall morphology of the microstructure. Using representative 3D models, we first quantify how the enforced minimum feature length reshapes the edge-length and face-size distributions and produces readily meshable polyhedral geometries. We then demonstrate that this geometric control translates directly into substantial gains in subsequent FE discretization quality and numerical stability, enabling large-scale cohesive-zone fracture simulations that would otherwise be computationally prohibitive or prone to premature numerical failure.

## 2. Methodology

In this section, we present the numerical modeling methodology employed in this study to enable efficient and reliable simulation of bioinspired composite microstructures. The description begins with the tessellation-based representation used to model the heterogeneous microstructure and highlights the resulting geometric features that pose significant challenges for subsequent volumetric meshing and cohesive-zone discretization. Building on this problem formulation, a geometric conditioning procedure is then introduced as a preprocessing step to systematically control undesirable small-scale features and produce simulation-ready polyhedral meshes. Together, these components define a consistent modeling workflow that directly supports the fracture simulations and parametric studies presented in the subsequent section.

### 2.1. Tessellation-Based Microstructural Modeling of Bioinspired Composites

Many bioinspired composites derive their exceptional mechanical behavior from architectures composed of a stiff “hard phase” such as mineral platelets, ceramic blocks, or other rigid inclusions embedded within a comparatively compliant organic matrix or interfacial layer. A natural and mathematically rigorous way to represent these heterogeneous architectures is through polyhedral grains generated by tessellation-based methods. In particular, Voronoi and Laguerre (or power) diagrams have emerged as powerful tools for constructing 3D polyhedral microstructures that capture key geometric attributes of biological materials.

Voronoi tessellations have been widely employed to reproduce nacre-inspired platelet arrangements as well as to investigate damage evolution, interface behavior, and failure mechanisms under various loading conditions [41,42,43]. Similar Voronoi-based representations have been used to model the extrafibrillar matrix (EFM) of bone [44,45,46], calcium plaques in human coronary arteries [47], and even the organization of epithelial tissues during wound closure processes [48]. These studies illustrate the versatility of Voronoi microstructures for mimicking systems in which stiff mineral or cellular units interact through compliant interfaces. Complementing these efforts, Laguerre tessellations have enabled the generation of hybrid composites featuring polydisperse inclusions and interface-rich behavior that better reflect natural mineralized composites. Such geometries have been used to explore the influence of interfacial properties on strain hardening, crack deflection, and toughening mechanisms in hybrid organic–inorganic nanocomposites [32]. The ability of Laguerre tessellations to explicitly incorporate particle size distributions provides a valuable extension for modeling systems in which platelet or grain sizes are non-uniform.

Together, these tessellation-based approaches provide realistic spatial variability, heterogeneous grain morphologies, and statistically meaningful size and shape distributions while maintaining physically consistent neighbor and interface connectivity. As a result, polyhedral representations derived from Voronoi or Laguerre diagrams form a robust and scalable foundation for FE and cohesive-zone simulations of diverse bioinspired composites, where the coupled behavior of stiff inclusions and compliant interfaces governs the emergent mechanical response.

### 2.2. Problematic Geometric Features and Computational Implications

Although Voronoi- and Laguerre-based polyhedral models provide a geometrically faithful representation of bioinspired microstructures, the raw tessellations generated from random seed distributions inherently contain problematic geometric features that hinder robust numerical simulations. The irregular spatial placement of seed points and the complex connectivity of 3D grain junctions routinely give rise to extremely short edges, narrow faces, and thin or sliver-like polyhedral cells. These undesirable features arise even under controlled sampling strategies and are a well-known byproduct of stochastic tessellation geometry.

**Figure 1:**
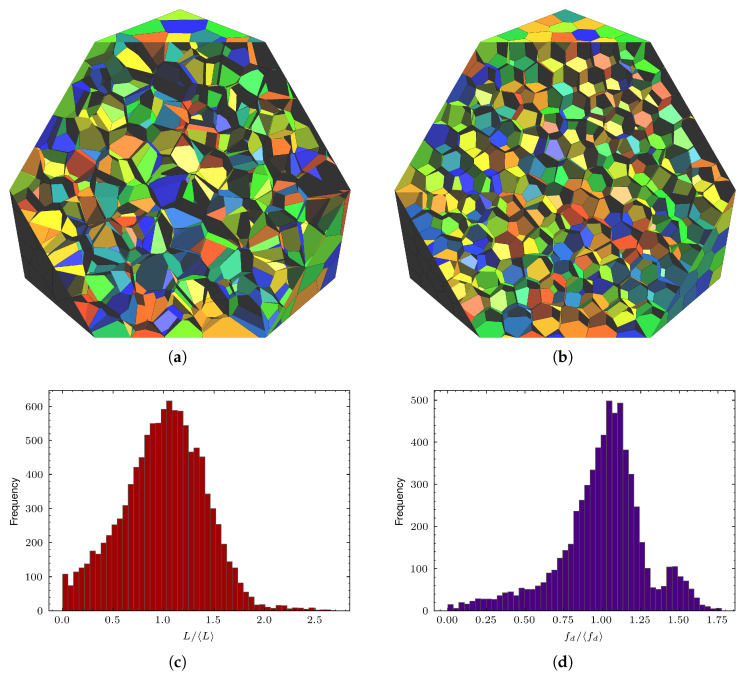
Effect of CVT regularization. Grain structures without any CVT regularization (**a**,**b**) with 200 CVT iterations. Edge-length (**c**) and face-diameter (**d**) distributions after 200 CVT iterations, where 〈L〉 and $\langle f_{d}\rangle$ denote the respective averages. A model with $10^{3}$ grains is used. Note the persistence of numerous small geometric entities even after extensive centroidal iterations.

Centroidal variants such as centroidal Voronoi tessellation (CVT) or centroidal Laguerre tessellation (CLT) are often employed in an attempt to improve cell regularity by iteratively relocating seed points toward their mass centroids. While such centroidal iterations can enhance overall grain compactness and reduce high-aspect-ratio distortions, they do not fully suppress the formation of unwanted small features. As illustrated in Figure 1, even hundreds of centroidal iterations leave a sizable lower-tail population of short edges and small faces, indicating that CVT/CLT regularization alone is insufficient for producing numerically robust polyhedral models.

Such geometric irregularities introduce several numerical challenges. Sliver-shaped features degrade FE mesh quality, leading to ill-conditioned stiffness matrices, reduced integration accuracy, and elevated sensitivity to numerical perturbations. In the case of simulations with CZM, small or highly distorted faces complicate the construction of cohesive interfaces, exacerbate artificial stress localization, and promote mesh-dependent crack trajectories. Moreover, the presence of extremely short edges or faces can trigger convergence issues in nonlinear solvers, necessitate excessive mesh refinement, and dramatically increase computational cost, which is particularly problematic when simulating large-scale polycrystalline or bioinspired architectures. Consequently, while raw tessellations capture microstructural realism, they remain suboptimal for high-fidelity FE modeling without careful preprocessing. These limitations motivate us to employ a dedicated regularization framework that can systematically eliminate problematic geometric features while preserving the essential topology and statistical morphology of the underlying grain structure.

### 2.3. Geometric Conditioning via Edge-Collapse Regularization

To obtain simulation-ready geometries without altering the intended microstructural morphology, we prescribe a geometric conditioning (regularization) step as a preprocessing operation on the conforming polyhedral mesh. The core implementation leverages a modified version of the edge-collapse functionality available in OpenFOAM (notably, the collapseEdges utility) [49], adapted for compatibility with the tessellation-based polyhedral mesh representation employed here, which performs local topology-preserving simplifications by merging vertices of short edges (and optionally faces) and updating the adjacent connectivity. In the present work, this operation is employed as a controlled mechanism to remove small geometric features that cannot be reliably resolved by the targeted FE discretization while still maintaining a conforming polyhedral grain partition suitable for subsequent volumetric meshing and insertion of cohesive elements along grain boundaries. Because the procedure operates directly on the connectivity and geometry of a general conforming polyhedral mesh, it applies equally to Voronoi- and Laguerre-based tessellations, including multi-phase or polydisperse microstructures with large variations in grain size.

The regularization is governed by a single user-defined minimum feature length, denoted by $l_{\min}^{\left(0\right)}$. Operationally, $l_{\min}^{\left(0\right)}$ sets a geometric resolution threshold: edges with length l(e) below this prescribed scale are considered undesirable for the intended mesh density, and are eligible for collapse. Because collapsing a short edge necessarily modifies the incident faces and cells, this single length scale also indirectly controls the elimination of small faces that are supported by such edges, thereby suppressing sliver-like facets that otherwise seed degenerate tetrahedra after volumetric meshing. In this sense, $l_{\min}^{\left(0\right)}$ is not merely a numerical parameter, but a physically interpretable modeling knob that links the geometric fidelity of the polyhedral microstructure to the intended FE resolution. Choosing a larger $l_{\min}^{\left(0\right)}$ increases the aggressiveness of feature removal, yielding a coarser but more robust geometry, whereas smaller $l_{\min}^{\left(0\right)}$ retains finer details at the cost of increased meshing difficulty and potentially severe time step restrictions in the explicit dynamics. In practice, a convenient guideline is to select $l_{\min}^{\left(0\right)}$ on the order of the target tetrahedral mesh size *h* used in the downstream FE discretization so that geometric entities below the intended numerical resolution are eliminated prior to meshing. Following this rationale, we set $l_{\min}^{\left(0\right)}=0.5\;\mathrm{mm}$ in the simulations of Section 3.2 in order to match the global target mesh size adopted for volumetric meshing.

During conditioning, each candidate collapse is executed only if the local modification preserves a valid non-inverted conforming mesh and does not introduce new undesirable features in the affected neighborhood. In practice, these safeguards correspond to enforcing positive cell volumes and limiting excessive local geometric distortion in the updated connectivity, consistent with the requirements of downstream FE meshing and cohesive-zone discretization. More specifically, each proposed collapse is accepted only if the updated local connectivity remains admissible, i.e., it does not create inverted polyhedra, invalid non-manifold configurations, or inconsistent face ownership across adjacent cells. Any locally degenerate entities introduced by a collapse (e.g., zero-length edges or zero-area facets arising from redundant vertices) are immediately eliminated via local cleanup and connectivity updates, ensuring that the resulting mesh remains a valid conforming partition suitable for robust FE meshing and cohesive-interface insertion. Therefore, the resulting regularized polyhedral mesh exhibits a truncated lower tail in the edge-length and face-diameter distributions, substantially reducing the likelihood of extremely small tetrahedra and improving the element shape statistics in the subsequent volumetric mesh. Furthermore, the procedure can be used in settings that require boundary constraints, such as periodic homogenization by assigning protected priority levels to periodic boundary vertices/edges, thereby preventing collapse operations that would disrupt periodic face/edge matching. In this manner, edge collapses are restricted to the interior, while periodicity of the boundary pairing is preserved. These effects directly motivate the parametric study in Section 3, in which $l_{\min}^{\left(0\right)}$ is varied in order to quantify how the prescribed minimum feature size influences the mesh complexity, numerical robustness, and efficiency of cohesive fracture simulations of bioinspired composite microstructures. The overall geometric conditioning workflow adopted in this study is summarized in Algorithm 1 for clarity, highlighting the key preprocessing steps that connect tessellation generation to robust FE meshing and cohesive-zone simulation.
**Algorithm 1** Workflow summary for regularization-enabled fracture simulation1: Generate a conforming polyhedral tessellation (Voronoi or Laguerre) representing the composite microstructure.2: Apply centroidal relaxation (e.g., CVT/CLT updates) to remove large-scale geometric irregularities.3: Prescribe a minimum admissible feature length $l_{\min}^{\left(0\right)}$ consistent with the target FE mesh resolution.4: Identify polyhedral edges with $l\left(e\right)<l_{\min}^{\left(0\right)}$ as candidates for geometric regularization.5: Perform local topology-preserving edge collapse operations subject to admissibility checks (non-inversion, valid connectivity, boundary constraints).6: Update local connectivity and remove any degenerate geometric entities introduced by accepted collapses.7: Generate a volumetric tetrahedral mesh of the regularized geometry and insert cohesive elements along grain boundaries.8: Perform finite element simulation with cohesive-zone modeling under the prescribed loading conditions.


## 3. Results

To demonstrate the necessity and effectiveness of the prescribed geometric regularization step, we consider a representative 3D cuboid domain ($20\times 20\times 20\;{\mathrm{mm}}^{3}$) containing $10^{3}$ polyhedral cells that represent grain microstructure. Although the modeling workflow applies to larger and more complex geometries, this configuration provides a controlled yet sufficiently challenging setting in which the influence of regularization on which to clearly isolate and assess (i) geometric feature statistics and (ii) downstream FE meshing and fracture simulations. As shown in Figure 1a, raw tessellations generated from random sampling produce highly irregular grain geometries that are unsuitable for robust FE discretization. To remove this confounding factor, the tessellation is first subjected to 200 iterations of centroidal Voronoi relaxation, yielding grains that appear smooth and well-distributed at the cell scale (see Figure 1b).

Despite this apparent grain-scale regularity, closer inspection of lower-dimensional features reveals that a substantial population of very short edges and small faces persists even after extensive CVT processing (Figure 1c,d). These problematic features are inherent to tessellation-based constructions; they continue to hinder subsequent volumetric meshing, and complicate the insertion of cohesive interfaces along grain boundaries. In what follows, the regularization procedure described in Section 2.3 is applied to this CVT-processed tessellation. In addition, the prescribed minimum feature length $l_{\min}^{\left(0\right)}$ is varied in order to examine how controlling the smallest retained geometric scale reshapes the edge-length and face-diameter distributions (Section 3.1). We then demonstrate how this geometric control translates into practical gains in mesh quality, numerical robustness, and computational efficiency for interfacial fracture simulation of a bioinspired organic–inorganic composite using CZM (Section 3.2).

### 3.1. Effect of Regularization on Edge and Face Diameter Distributions

As detailed in Section 2.3, the prescribed regularization step enforces a user-defined minimum feature length $l_{\min}^{\left(0\right)}$ by collapsing edges with length $l\left(e\right)<l_{\min}^{\left(0\right)}$. This threshold defines the smallest geometric scale retained in the regularized polyhedral microstructure, and is selected in relation to the intended FE discretization. Because edge collapses locally modify the incident faces and cells, enforcing $l_{\min}^{\left(0\right)}$ also suppresses small or sliver-like faces supported by extremely short edges, thereby improving the overall geometric regularity of the grain shapes.

**Figure 2:**
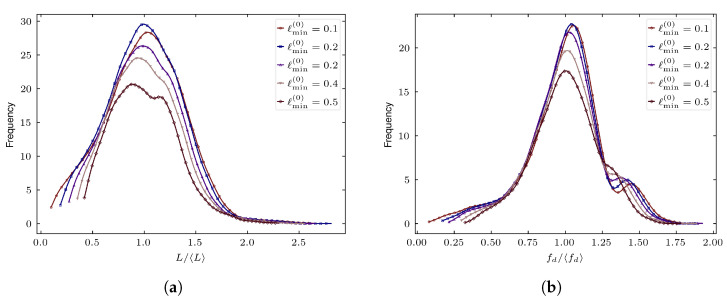
Effect of regularization for several values of the desired minimum feature size $l_{\min}^{\left(0\right)}$. Increasing $l_{\min}^{\left(0\right)}$ progressively eliminates short edges (**a**) and small faces (**b**), resulting in smoother and more right-shifted distributions.

Figure 2 illustrates the evolution of the normalized edge-length and face-diameter distributions as $l_{\min}^{\left(0\right)}$ is increased. Raising $l_{\min}^{\left(0\right)}$ sharply truncates the lower tail of the edge-length distribution, consistent with the prescribed elimination of geometric features below the target minimum scale. The face-diameter distribution exhibits a corresponding reduction in its lower tail, reflecting the indirect removal of small faces through local edge collapse updates. In addition to eliminating undesirable features, both distributions progressively shift toward larger normalized values and become more concentrated, indicating reduced geometric variability and more uniform grain shapes. For larger $l_{\min}^{\left(0\right)}$, the suppression of thin polyhedral features and sliver-like faces becomes more pronounced, producing tessellations that are better aligned with practical meshing requirements.

Together, these results demonstrate that the prescribed regularization step not only eliminates problematic small features but also enforces a consistent geometric length scale while markedly improving overall shape quality. The resulting meshes enable stable insertion of cohesive interfaces, are substantially easier to mesh with subsequent finite elements, and exhibit improved conditioning for nonlinear FE solvers, making them well suited for large-scale fracture simulations of bioinspired composites.

### 3.2. Regularization-Enabled Fracture Simulation of a Bioinspired Composite

**Figure 3:**
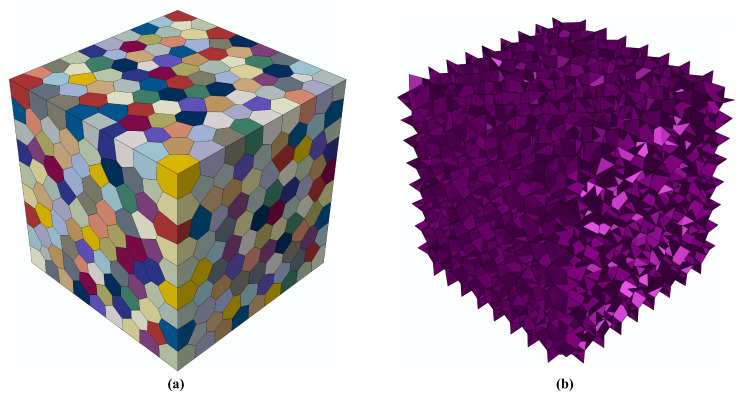
Geometric model of the bioinspired organic–inorganic composite: (**a**) polyhedral grains representing the inorganic hard phase and (**b**) interfacial cohesive zones inserted along grain boundaries to model the soft organic phase.

To evaluate the practical impact of the prescribed geometric regularization on a challenging interfacial fracture problem, we consider the CVT-relaxed tessellated cuboid domain introduced in this section and model it as a bioinspired organic–inorganic composite. Each polyhedral cell represents a grain of the stiff inorganic phase (Figure 3a), while the compliant organic phase is introduced by inserting cohesive interfaces along all grain boundaries (Figure 3b). This representation captures a broad class of bioinspired and polycrystalline composites in which macroscopic nonlinearity and failure are primarily governed by interfacial deformation and sliding mechanisms.

The inorganic grains and organic interfaces are discretized using linear tetrahedral elements (C3D4) and 3D cohesive elements (COH3D6), respectively, which are generated using the advanced free-meshing algorithm in Abaqus/CAE [50]. The hard phase is modeled as a linear elastic solid with Young’s modulus E=100GPa, Poisson’s ratio ν=0.28, and density $\rho =3000\;\mathrm{kg}/m^{3}$. Interfacial behavior of the soft organic phase is described using a bilinear cohesive traction–separation law. Prior to damage, we employ an uncoupled linear elastic response with penalty stiffness $k_{nn}=k_{ss}=k_{tt}=3.694\times 10^{5}\;\mathrm{MPa}/\mathrm{mm}$ and zero coupling terms $k_{ns}=k_{nt}=k_{st}=0$. The penalty stiffness is selected based on the mesh size and surrounding tablet properties; a density $\bar{\rho }=7.93\times 10^{-10}\;\mathrm{tonne}/{\mathrm{mm}}^{2}$ is used together with the penalty stiffness to obtain an optimal stable time increment Δt for Abaqus/Explicit. Damage initiation follows the quadratic nominal stress criterion [51] with cohesive strength 50MPa, and post-initiation degradation is described using a mode-independent energy-based linear damage evolution with target fracture energy $0.5\;\mathrm{kJ}/m^{2}$ [32,43]. A small viscosity coefficient of $1\times 10^{-4}$ is introduced to numerically stabilize softening. We select a bilinear cohesive law because it provides a robust and widely used representation of interfacial failure in bioinspired composites with well-defined strength and fracture energy parameters; importantly, the main conclusions of this work are not tied to the specific softening shape (e.g., bilinear versus exponential or polynomial laws), since geometric regularization acts as a constitutive-independent preprocessing step that eliminates undesirable small geometric entities responsible for severe mesh distortion and prohibitively small stable time increments in explicit analysis. The simulations impose quasi-static uniaxial compression by prescribing vertical displacements on the top surface (Y=20mm) while fixing the bottom surface (Y=0) to achieve 8% compressive strain, with traction-free lateral boundaries, and are conducted using Abaqus/Explicit to capture interfacial damage, softening, and contact interactions. Although the present results focus on compression, the role of regularization is expected to remain equally critical for tensile loading, dynamic impact, and cyclic/fatigue simulations, since the underlying numerical bottleneck arises from tessellation-induced geometric degeneracies rather than from the particular loading mode.

**Figure 4:**
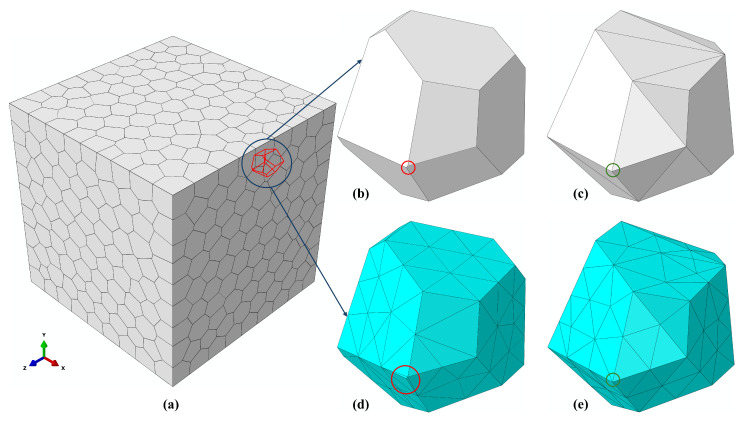
Impact of geometric regularization on volumetric meshing: (**a**) representative polyhedral grain geometry, (**b**) extremely short edge in the non-regularized grain, (**c**) elimination of the short edge after regularization, (**d**) severely distorted tetrahedral element generated from the non-regularized geometry, (**e**) high-quality tetrahedral elements obtained after regularization.

For this class of models, volumetric meshing of polyhedral grains constitutes one of the most critical and failure-prone stages of the simulation pipeline. In the absence of geometric regularization, even CVT-relaxed tessellations retain a substantial number of extremely short edges and small faces (Figure 1c,d). When passed to a free-meshing algorithm, these problematic geometric features inevitably lead to highly distorted tetrahedral elements. As illustrated in Figure 4b,d, meshing a non-regularized grain produces elements with aspect ratios exceeding 3000 and normalized shape factors as low as $10^{-7}$, even under aggressive mesh refinement. In Abaqus/CAE, the aspect ratio of an element is defined as the ratio between the longest and shortest edges of the element; large values indicate highly distorted geometry. The normalized shape factor of a tetrahedral element is defined as the ratio of its volume to the volume of an equilateral tetrahedron having the same circumradius; values near 1 indicate optimal element shape, whereas values approaching 0 indicate degeneracy. For the model with $10^{3}$ cells considered here, the non-regularized geometry requires more than $4.4\times 10^{5}$ tetrahedral elements and $9\times 10^{4}$ cohesive elements merely to avoid immediate meshing failure. Despite this extreme discretization, the resulting mesh requires an explicit stable time increment on the order of $10^{-13}\;s$ (Table 1), taking days to simulate up to approximately 0.5% compressive strain, and still diverges due to severe element distortion during loading. These results demonstrate that without geometric regularization, tessellation-based microstructures are often impractical for modeling and simulation of bioinspired composites, leading to prohibitively high computational costs or premature numerical failure.

In contrast to the non-regularized case, enforcing geometric regularization prior to FE meshing removes all lower-dimensional features below the prescribed threshold, yielding a substantially simplified grain morphology. For the present model, selecting a target mesh size of 0.5 mm and enforcing $l_{\min}^{\left(0\right)}=0.5$ eliminates every short edge less than this set threshold, resulting in very high-quality tetrahedral elements (Figure 4c,e) that would otherwise induce severe element distortion (Figure 4d). As summarized in Table 1, the same 10^3^-cell microstructure can then be discretized using approximately $1.0\times 10^{5}$ tetrahedral elements and $2.7\times 10^{4}$ cohesive elements, for a nearly fivefold reduction in total element count while maintaining uniformly high element quality. The resulting explicit simulation requires a stable time increment of ∼10^−8^ s and converges smoothly to the prescribed 8% compressive strain within approximately 150 s on a consumer desktop. This comparison demonstrates that geometric regularization transforms an otherwise intractable fracture analysis into a computationally feasible and numerically robust simulation.

**Figure 5:**
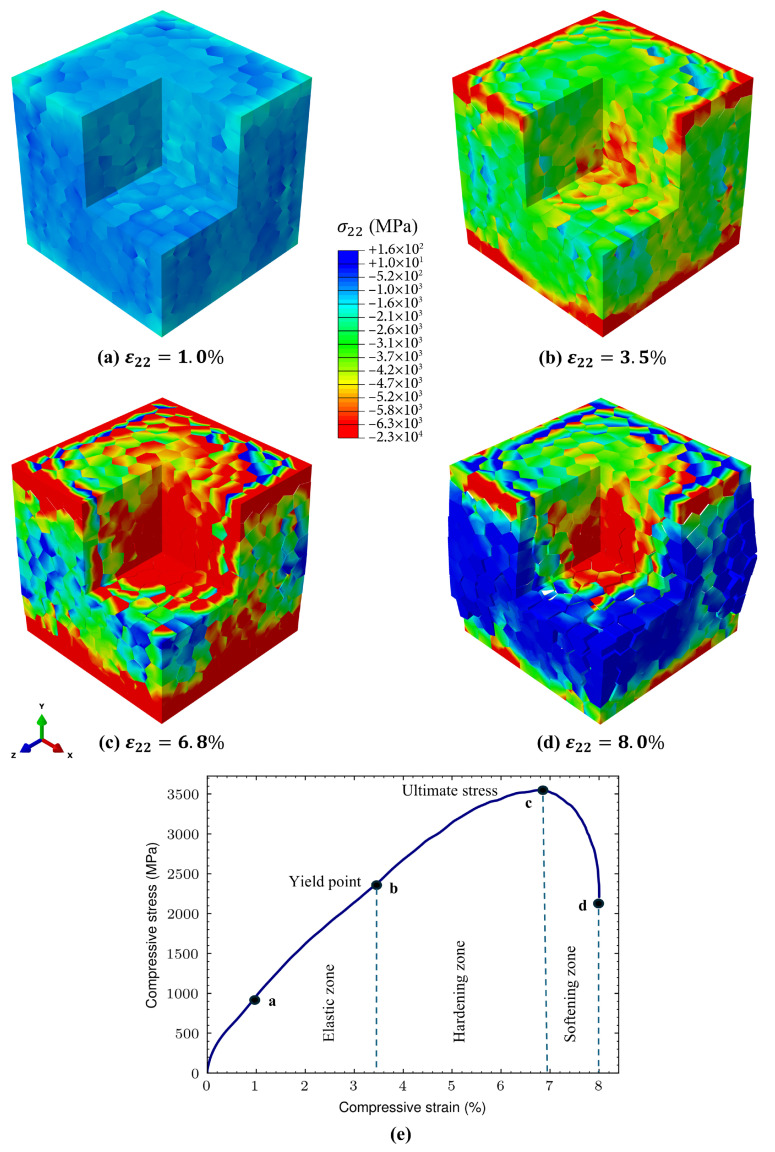
Fracture simulation results for the regularized model under uniaxial compression: (**a**–**d**) snapshots of the axial stress component $\sigma_{22}$, illustrating progressive deformation and interfacial cracking; (**e**) macroscopic stress–strain response exhibiting a near-linear elastic regime, a post-yield nonlinear regime governed by cohesive damage and energy dissipation, and a final softening regime associated with crack coalescence and loss of load-carrying capacity.

The converged fracture response enabled by the regularized model is shown in Figure 5. The axial stress fields $\sigma_{22}$ (Figure 5a–d) reveal progressive stress localization and redistribution as loading proceeds. Owing to the strong stiffness contrast between phases, compressive stresses within the mineral grains reach the multi-gigapascal range (Figure 5c), substantially exceeding the cohesive strength of the organic interfaces. This mismatch promotes the initiation of damage and progressive cracking along grain boundaries, accompanied by local stress relaxation in regions undergoing interfacial damage. Beyond the ultimate stress point, the accumulation and interaction of cracked interfaces lead to a gradual loss of load-carrying capacity, as reflected in the softening portion of the macroscopic response.

**Figure 6:**
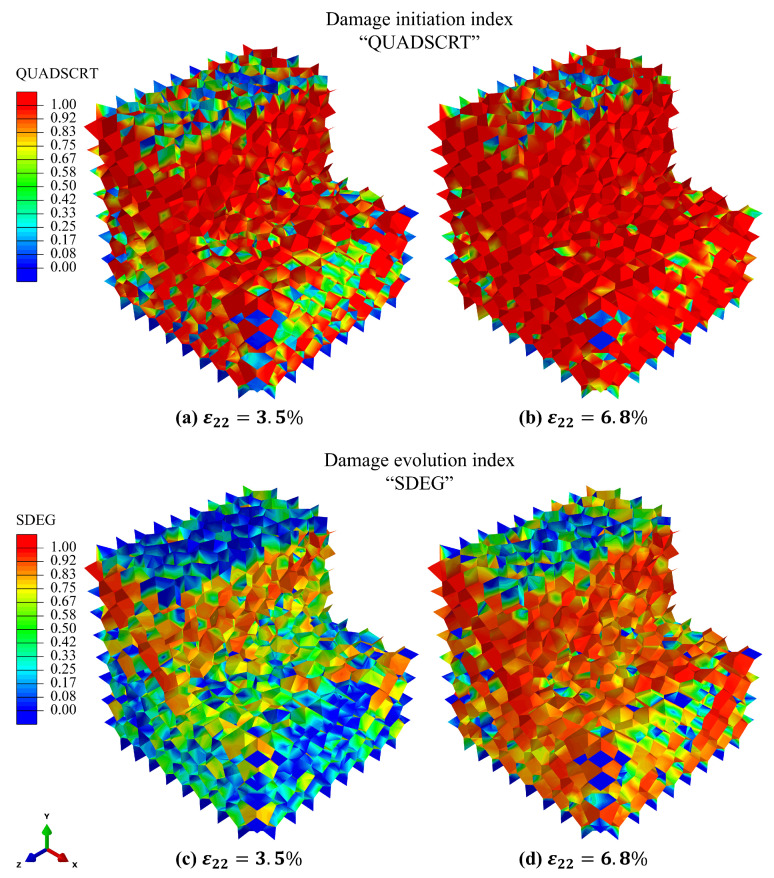
Interfacial damage evolution during compression: (**a**,**b**) QUADSCRT distributions indicating the onset and spatial spread of damage initiation across cohesive interfaces; (**c**,**d**) SDEG distributions showing progressive degradation and non-localized damage evolution within the adhesive phase.

The macroscopic stress–strain curve in Figure 5e can be interpreted in terms of the underlying interfacial damage mechanisms revealed in Figure 6. The initial near-linear regime corresponds to predominantly elastic deformation of the hard mineral phase with negligible interfacial damage. As loading increases, a post-yield nonlinear regime emerges in which a growing fraction of cohesive interfaces satisfies the damage initiation criterion, as indicated by the expanding QUADSCRT field (Figure 6a,b). The spatially distributed nature of this damage initiation promotes progressive energy dissipation across numerous interfaces rather than localized failure, giving rise to an extended nonlinear hardening-like response. At larger strains, continued damage evolution and degradation of cohesive stiffness are captured by the SDEG field (Figure 6c,d), leading to coalescence of cracked interfaces. This transition marks the onset of macroscopic softening and culminates in failure at a bulk strain of $\epsilon_{\mathrm{failure}}^{\mathrm{bulk}}\approx 8.0\%$.

Taken together, these results demonstrate that geometric regularization is not merely a preprocessing convenience but a critical enabler of successful modeling and complex cohesive-zone simulations of tessellation-based bioinspired composites. By enforcing a consistent geometric length scale and eliminating problematic lower-dimensional features, regularization yields meshable geometries, well-conditioned discretizations, and stable numerical solutions that allow the fracture process to be analyzed and interpreted in a physically meaningful manner.

## 4. Conclusions

This work demonstrates that geometric regularization is a critical enabler for efficient and robust FE simulation of bioinspired organic–inorganic composites constructed from tessellation-based polyhedral models. Although Voronoi- and Laguerre-based tessellations provide physically meaningful grain morphologies, their raw geometries inherently contain lower-dimensional degeneracies such as extremely short edges and small or sliver-like faces. These impede volumetric meshing, destabilize cohesive-interface insertion, and often render large-scale fracture simulations impractical. By prescribing a minimum admissible geometric feature length prior to FE meshing, the employed regularization step systematically removes these problematic entities through local topology-preserving operations while preserving conforming grain connectivity and the statistical morphology of the microstructure. The resulting geometries exhibit sharply improved edge-size and face-size distributions, uniformly high element quality, and well-conditioned discretizations, directly translating into dramatic gains in numerical stability and computational efficiency. Application to a 3D bioinspired composite model shows that regularization reduces element counts by several factors, increases stable explicit time increments by multiple orders of magnitude, and enables smooth resolution of progressive interfacial damage, crack evolution, and macroscopic stress–strain response under compressive loading. These results establish geometric regularization as being not merely a preprocessing convenience but a necessary foundation for scalable and high-fidelity cohesive-zone simulations of complex tessellation-based composite materials.

## Figures and Tables

**Table 1:** Influence of geometric regularization on discretization quality and explicit solver performance.

Metric	Without Regularization	With Regularization
No. of tetrahedral elements	445,908	101,221
No. of cohesive elements	90,058	27,599
Worst aspect ratio	2538	127.8
Worst shape factor	$5.46\times 10^{-7}$	$1.27\times 10^{-3}$
Stable time increment (s)	$1.03\times 10^{-13}$	$9.02\times 10^{-9}$
Convergence status	Diverged	Converged

## Data Availability

All data generated or analyzed during this study are included in this article. A Docker image of the implementation of the regularizer program is hosted at https://github.com/Rumi381/regularizer.git (accessed on 10 February 2026).

## Abbreviations

The following abbreviations are used in this manuscript:

3D	Three-dimensional
FE	Finite Element
FEM	Finite Element Method
CZM	Cohesive Zone Method
CVT	Centroidal Voronoi Tessellation
CLT	Centroidal Laguerre Tessellation
